# Behavioural adaptations to flight into thin air

**DOI:** 10.1098/rsbl.2016.0432

**Published:** 2016-10

**Authors:** Sherub Sherub, Gil Bohrer, Martin Wikelski, Rolf Weinzierl

**Affiliations:** 1 Ugyen Wangchuck Institute for Conservation and Environment Research, Bumthang, Bhutan; 2 Department of Migration and Immuno-Ecology, Max Planck Institute for Ornithology, 78315 Radolfzell, Germany; 3 Department of Biology, University of Konstanz, 87457 Konstanz, Germany; 4 Department of Civil, Environmental and Geodetic Engineering, Ohio State University, Columbus, OH 43210, USA

**Keywords:** movement ecology, thermalling, aerodynamic, non-powered flight

## Abstract

Soaring raptors can fly at high altitudes of up to 9000 m. The behavioural adjustments to high-altitude flights are largely unknown. We studied thermalling flights of Himalayan vultures (*Gyps himalayensis*) from 50 to 6500 m above sea level, a twofold range of air densities. To create the necessary lift to support the same weight and maintain soaring flight in thin air birds might modify lift coefficient by biophysical changes, such as wing posture and increasing the power expenditure. Alternatively, they can change their flight characteristics. We show that vultures use the latter and increase circle radius by 35% and airspeed by 21% over their flight altitude range. These simple behavioural adjustments enable vultures to move seamlessly during their annual migrations over the Himalaya without increasing energy output for flight at high elevations.

## 1. Introduction

Migrant birds often spend part of their lives at low altitudes and then ascend to altitudes of up to 9000 m [1]. Lift acceleration is proportional to air density, and thus, when keeping all other parameters constant (such as flight speed, lift coefficient, wing area), will decrease with elevation (equation (2.1)), whereas the gravitational force is near constant. Birds need to adapt their flight-related biophysical properties and/or flight behaviour to thin air. While the physiological adaptations of flying at extreme altitudes are partially understood, particularly with regard to the oxygen transport capacity of blood haemoglobin [2], the behavioural adaptations of high-flying birds are much less known [3].

Bar-headed geese (*Anser indicus*) cross the Himalayan mountains, using a ‘roller-coaster’ flapping-flight strategy [4]. Hummingbirds in wind tunnel experiments flap faster with larger amplitude and increase power expenditure to increase their lift coefficient in thin air [5]. In contrast, obligate soaring birds, such as vultures, are largely unable to employ a powered flight mode for long durations [6].

Himalayan vultures are among the heaviest flying bird species with large wingspan (7.9 kg, 2.4 m, respectively, on average, in our study). We assess the birds' strategies of thermalling from 50 to 6500 m altitude, while crossing the Himalaya during their natural annual flights.

Generally, a thermalling bird with non-powered flight has two contrasting options to create the lift necessary to maintain upward acceleration in a rising thermal in thinner air: (i) biophysical—change its wing or feather posture and inclination to increase the lift coefficient; (ii) behavioural—change its flight characteristics, i.e. increase its thermalling radius and/or flight speed. We leverage a new analysis approach to determine wind speed and, consequently, airspeed from high-frequency GPS data of thermalling vultures, and provide direct observations of the strategy employed by free-flying wild vultures soaring over the Himalaya.

## 2. Methods

### (a) Bird capture and data recording

Twenty-one birds were captured in Bhutan between May 2014 and February 2015 using a wire-mesh cage trap (6 × 6 × 40 feet) and fitted with GPS data loggers (45 g, cellphone link, e-*obs* GmbH), using an approximately 30 g Teflon–nylon harness. Daily (02. 00–20.00 GMT), loggers were set to periodically collect 1 Hz GPS fixes for 10 min, whenever solar charge allowed. We collected 1 694 828 such GPS fixes between 1 August 2014 and 3 July 2015. Data are available through the Movebank archive [7].

### (b) Background aerodynamic theory

The lift coefficient, *C*_L_, is determined by the combined effects of all lift-generating mechanical properties of a gliding bird, such as body shape, size, wing posture and angle of attack. At balanced flight2.1
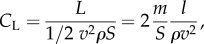
where *ρ* is air density, *v* airspeed, *L* lift force, *S* wing area, *m* bird mass, *m*/*S* wing loading; and *l* = *L*/*m* lift acceleration (lift force per unit mass).

For a bird flying in circles, as the vultures do while thermalling, we can write the balance of forces in two dimensions: vertical—balancing gravity (equation (2.2)) and tangential—balancing the centripetal force (equation (2.3))2.2
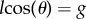
and2.3
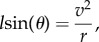
where *θ* is banking angle, and *r* is thermalling circle radius. In order to stay aloft during circling flight, the bird needs to generate a lift acceleration of2.4
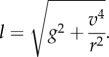
Each of the terms in equations (2.2)–(2.4) can be determined from the flight data and annotated environmental information and allows us to determine which of the terms trades off with decreased air density. Realistic estimates of air density during the observed flight were obtained from the European Centre for Medium-Range Weather Forecasts (ECMWF) ERA-Interim weather reanalysis dataset and annotated to the tracks using the Movebank-Env-DATA service [8,9]. Wind speed and direction can be determined from the flight path of the bird during circling flight using the analysis package developed by Weinzierl *et al.* (2016, part of the R-package ‘move’, https://cran.r-project.org/web/packages/move/index.html) (for formulation of the method see electronic supplementary material, appendix 1). This method expands the approach by Treep *et al.* [10]. It assumes that wind speed causes horizontal displacement during thermal soaring. Assuming that over a short time and space the variation of horizontal wind speed is small, the amount of distortion of each ‘loop’ within a thermalling flight pattern can be used to determine the mean wind speed and direction within the area enclosed by each thermalling loop. The wind speed is then subtracted from the GPS ground-speed measurements to determine the bird's airspeed.

### (c) Data analysis

We estimated the bird's mean airspeed, *v* for 30 s intervals. Only those sequences where the bird made a full circle within 30 s were used. Assuming that within a single thermalling circle the bird made a balanced turn at a constant airspeed, a constant circle radius and a constant bank angle, we calculated the angular rate2.5
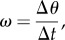
where Δ*θ* is the cumulative angular difference across 31 fixes and Δ*t*= 30 s; the circle radius *r* = *v*/*ω*, where *v* is airspeed (m s^−1^) and the lift acceleration2.6
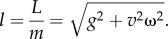


We fixed wing loading of *m*/*S* = 10.54 ± 0.9 kg m^−2^, based on our measurements of 26 individuals. Because the birds' mass changes significantly as they feed, we use the population mean rather than use an individually observed value. We further assume that the horizontal wind speed is small relative to the airspeed of the circling bird, and ignore altitudinal variation in gravity and buoyancy. The lift coefficient associated with each non-overlapping track segment can be determined as2.7
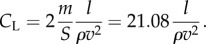
Track segments for which no ECMWF data could be obtained were dropped, resulting in a final sample size of *n* = 8595. Processed wind data per circle and observed physiological data are provided in the electronic supplementary material, appendix 2.

## 3. Results

**Figure 1.**
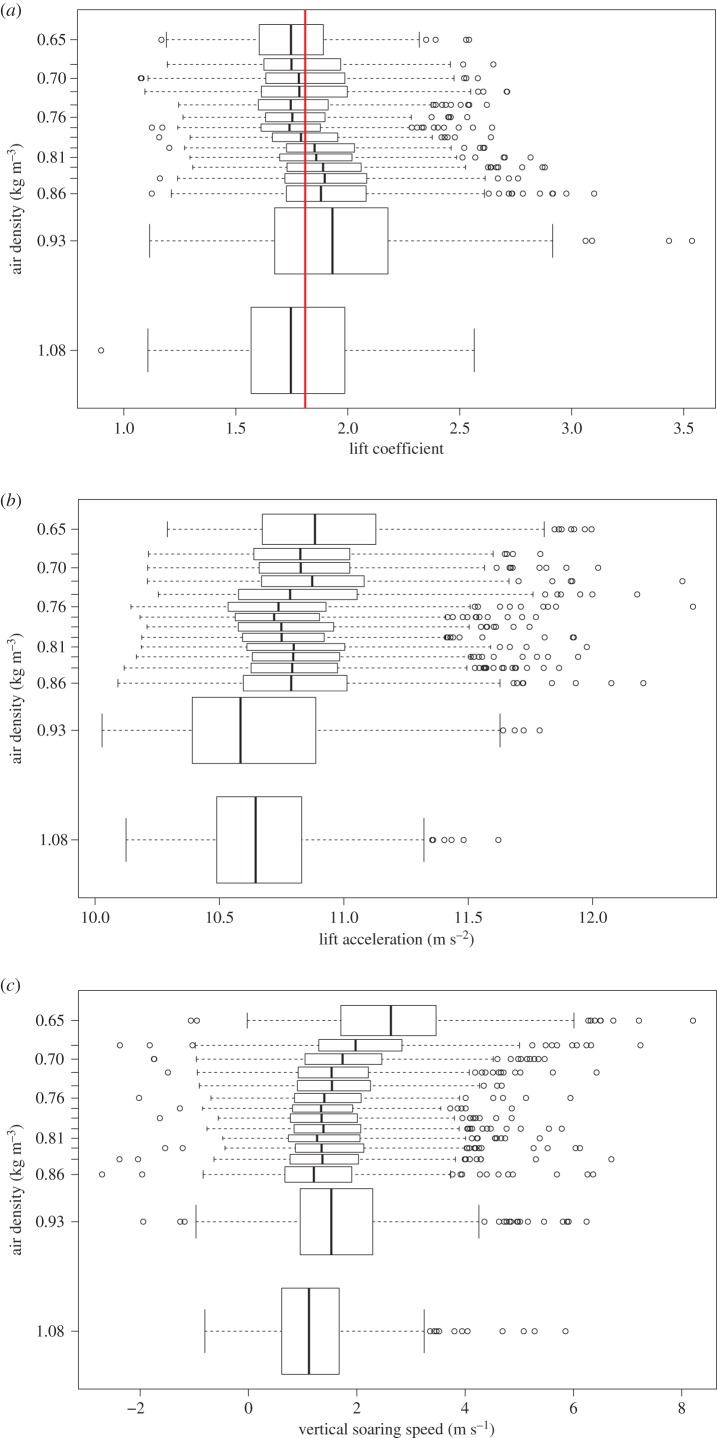
Characteristics of acceleration at increasing elevations. (*a*) The vultures show small variations (−4% to +7%) in the lift coefficient over the entire flight elevation (air densities) range (red line marks the overall mean); (*b*) the lift acceleration varied very little with height (less than 2%); (*c*) the vertical soaring speed remained near constant, and even increased above 4500 m (air density less than 0.7 kg m^−3^). Boxes show mean (vertical line), quartile (box) and 95% (whiskers) confidence interval, as well as outliers (circles) in 15 elevation bins with equal observation numbers.

Air densities varied almost twofold between 50 and 6500 m flight altitude. We found that the lift coefficient and lift acceleration remained relatively constant throughout the entire range of elevations (figure 1*a*,*b*). While in theory the wing area could have changed to perfectly offset the effects of the angle of attack on the lift coefficient, a more parsimonious explanation is that body posture remained near constant.

For the purpose of analysis and comparison of flight behaviour in low versus high altitudes, we define a subsample of high-flying and low-flying groups (5% of observed points, *n* = 430, at lowest and highest air density, respectively). Mean air density in the low-flying group (*ρ*_l_ = 1.099 kg m^−3^) is 70.05% higher than for the high-flying group (*ρ*_h_ = 0.646 kg m^−3^). The lift coefficients, however, are very similar between high-flying (*C*_H_ = 1.74) and low-flying (*C*_L_ = 1.83) groups (4.87% difference).

**Figure 2.**
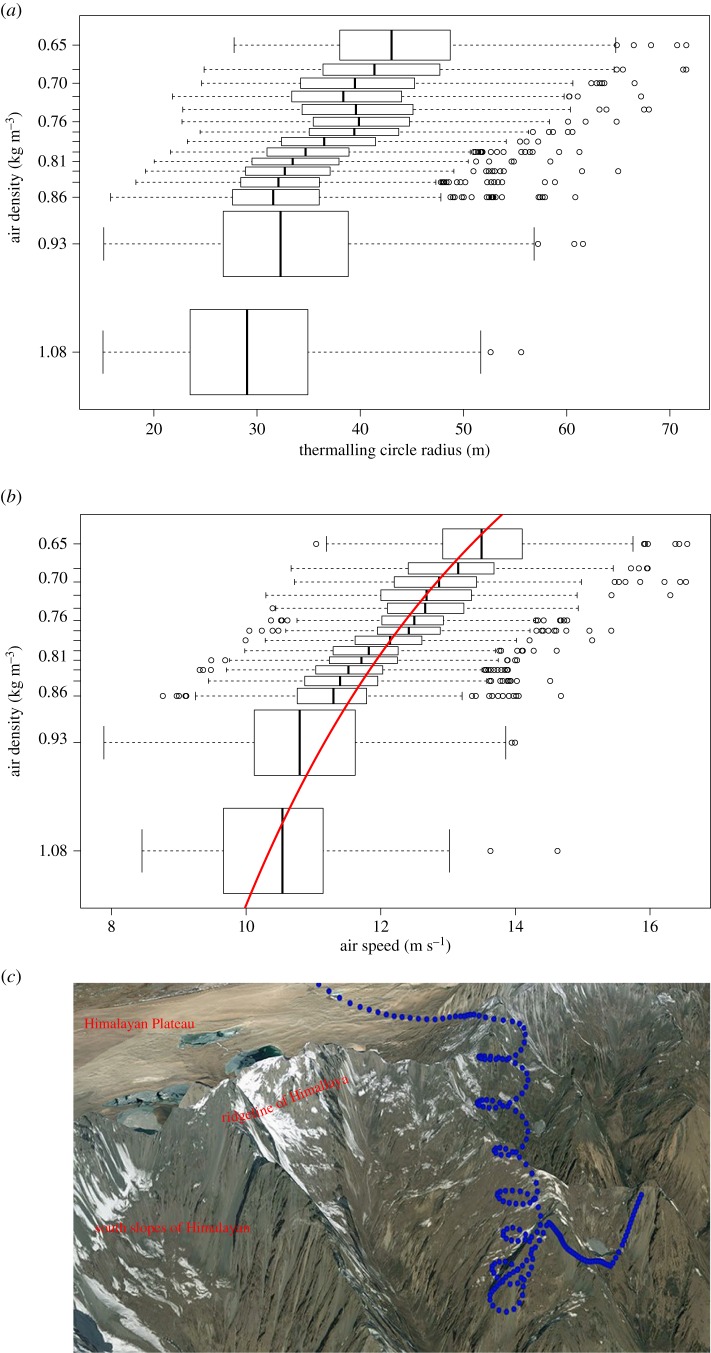
Behavioural changes in the flight characteristics of thermalling vultures. (*a*) Flight circle radius increases in relation to decreasing air density (increasing elevation). (*b*) Airspeed increases with elevation. Red line represents a linear model fitted to the inverse of the air density and the squared airspeed (slope = 115.7, *R*^2^ = 0.46, *p* < 0.0001). Data show average (vertical line), quartile (box) and 95% CI, as well as outliers (circles) in 15 elevation bins. (*c*) One flight segment of the vulture Yoezer. Dots mark the reported GPS locations of the vulture during flight over the Himalaya ridge. Land surface and topography obtained from GoogleEarth.

The birds increased their mean circle radius by *ca* 12.5% per 1000 m increase in altitude (figure 2*a*). The birds' airspeed increased strongly as they were ascending into thinner air (figure 2*b*). While vultures flying close to sea level flew at speeds of 10.5 m s^−1^, they sped up to 13.5 m s^−1^ at 6500 m altitude, an approximately 30% increase in airspeed.

## 4. Discussion

For soaring birds, the energetic cost of flying is very low [11]. We therefore assume they adjust and optimize their gliding flight to any altitude within their flight space by behavioural means other than increased power output.

We found that the lift coefficient of soaring Himalayan vultures changed very little despite a large change in air density (figure 1*a*). The relatively constant lift coefficient provides no evidence to support compensation for thinner air by biophysical changes to wing configuration, and by parsimony, suggests that they adjust for thinner air behaviourally by adjusting their flight characteristics, such as increasing their airspeed and circle radius.

By calculating the theoretical ratio between the corresponding velocities needed for maintaining a lift acceleration that will offset gravity at high and low elevations (using equation (2.1)), we can determine the airspeed compensation over the vultures' flight elevation range as4.1
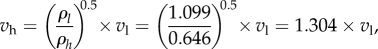
where *ρ*_l_, *ρ*_h_, *v*_l_, *v*_h_ are the air densities and airspeeds at low and high altitudes, respectively.

Assuming a fixed wing configuration, as indicated by the relatively constant lift coefficient, the airspeed compensation predicted by equation (4.1) will be needed to keep the sink rate constant while flying in a straight line in air thinned from 1.099 to 0.646 kg m^−3^. The magnitude of the observed airspeed difference between low and high elevations—roughly one-third (figure 2*b*)—is in agreement with this expected value (30.4%).

Soaring in a circular flight pattern provides additional constraints as the banking angle diverts some of the lift force from countering gravity to countering the centripetal force. The sink rate of a turning bird, *v*_st_, can be corrected relative to the straight-line equilibrium sink rate, *v*_s_, following [12]: 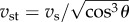. We found that low-flying birds fly at a shallow banking angle of *θ* = 23 ± 1.5°. Therefore, reducing the banking angle, which for a fixed velocity will translate to increasing the circling radius, can at best improve the sink rate (and therefore the vertical soaring speed) by a small amount (approx. 11%). The choice of banking angle at low elevations is thus driven by the needs of centring the thermal. However, at higher elevations, circles of the same size would translate to a steeper banking angle (approx. 42°, equation (2.3)) because of the faster flight velocity in thinner air, which impacts the sink rate by approximately 36%. We find that high-flying vultures increase circling radius by 56% (from 28.3 to 44.3 m) relative to low-flying ones, thus keeping banking angle as well as lift acceleration roughly constant throughout the elevation range of their flight.

The above calculations apply to theoretically ideal circling flight in a spatially constant wind field within an area-limited thermal. In reality, the circling radius should be driven, to a large degree, by the availability of thermal uplift, the size of the thermals and the distribution of vertical airspeeds within the thermal column. Little is known about the explicit distribution of size and structure of thermals over the Himalaya (or anywhere else) at any given time, though they are generally expected to be stronger near their centre and widen with elevation. Our calculations, supported by our observations, indicate that at high elevations the choice of thermalling circle radius is increasingly constrained by the air density in addition to the regular constraints of centring a thermal. Our finding that vertical soaring speed does not decrease, and in fact, slightly increases, with elevation (figure 1*c*) indicates that given the thermal uplift conditions in the Himalaya vultures employ behavioural adjustment to their flight characteristics that allow efficient soaring at high elevations despite the increasing challenges (figure 2*c*). In general, we expect that most soaring bird species, where individuals experience widely differing air densities, will use similar behavioural adaptations to thermalling flight in thin air (see also [13]), namely keep power output minimal and increase airspeed and circling radius.

## Supplementary Material

Appendix 1: Speed estimation method

## Supplementary Material

Appendix 2: (A).High resolution (1Hz) GPS track and associated movement data of Himalayan Vulture. (B). Wing area and weight data of Himalayan Vulture

## Data accessibility

Data are deposited in the Movebank Data Repository [7]: http://dx.doi.org/10.5441/001/1.143v2p2k.
