# Supplementary material for: Behavioural adaptations to flight into thin air
Source: Biol Lett. 2016 Oct;12(10):20160432. doi: 10.1098/rsbl.2016.0432 (PMC5095188; doi:10.1098/rsbl.2016.0432)
Supplement: Appendix 1: Speed estimation method [file rsbl20160432supp1.docx]

**Behavioural adaptations to flight into thin air** (Sherub Sherub^1,2,3,*^, Gil Bohrer^4^, Martin Wikelski^2,3^ & Rolf Weinzierl^2,3^)

### Appendix 1

### Formulation for estimation of wind speed and air speed from GPS-observed locations and ground speeds

Definitions:

$G=\left[ \vec{g'}_{1},\ldots,\vec{g'}_{n} \right]$ – Set of GPS observed ground-speed vectors (x (latitudinal) and y (longitudinal) components) [m/s].

$\Delta t$ - Constant sampling rate [s].

$\vec{w}=\left( w_{x},w_{y} \right)$ - Assumed constant horizontal wind vector [m/s] within a single thermal circle (here we defined a single circle over 30 seconds) (Figure 1).

$\vec{a}_{i}=\left( a_{i,x},a_{i,y} \right)\equiv\left( \vec{g_{i}}-\vec{w} \right)$ - The bird’s air-speed vector [m/s].

$a_{i}=\left\| \vec{a'}_{i} \right\|$ - The apparent air speed, $a_{i}$ [m/s], is defined using the vector-distance operator.

Approach:

Within each circle, we model $a_{i}$ as a first-order autoregressive process, AR(1), with mean, $a$, representing the bird's assumed constant air speed, and unexplained and small-scale variance due to turbulence , $\sigma_{a}^{2}$, such that $a_{i}=\left( 1-\phi\right)a+\phi a_{i-1}+N\left( \sigma_{a}^{2} \right)$, where *N* is a mean-zero Gaussian random distribution and $\phi$ $(0\leq\phi\leq1$), is an autocorrelation coefficient. Substituting the observed ground speed vector into the definition of air speed, we obtain:

$\vec{a}_{i}=\vec{g'}_{i}-N\left( \sigma_{g}^{2}I \right)-\vec{w}$ (1)

where and $I$ is the 2x2 identity matrix.

By reorganizing equation (1) and assuming that the GPS error $\sigma_{g}$ is small relative to *a_i_*, we obtain the following approximation for air speed, $\tilde{a_{i}}$

$a_{i}=\left\| \left( \vec{g'}_{i}-\vec{w} \right)-N\left( \sigma_{g}^{2}I \right) \right\|\approx\tilde{a_{i}}+N\left( \sigma_{g}^{2} \right)$. (2)

We can derive the negative log likelihood of observing the sequence of GPS groundspeed vectors, $G$, given wind $\vec{w}$as

$\begin{aligned} l\left( G,\vec{w} \right)=-log\left( P\left( \tilde{\alpha_{1}} \right)\prod_{i=2}^{n} P\left( \tilde{a_{i}}\vee\tilde{a_{i-1}} \right) \right)= \\ \frac{1}{2}\left\{ nlog\left( \sigma^{2} \right)-log\left( 1-\phi^{2} \right)+\frac{1}{\sigma^{2}}\left[ \left( \tilde{\alpha_{1}}-a \right)^{2}\left( 1-\phi^{2} \right)+\sum_{i=2}^{n} \left( \tilde{\alpha_{i}}-\left[ \left( 1-\phi\right)a+\phi\tilde{\alpha_{i-1}} \right] \right)^{2} \right] \right\} \end{aligned}$ (3)

where constant terms were omitted. By setting the derivative of the likelihood function with respect to zero, we obtain the maximum likelihood estimator for mean air speed

$\hat{a}=\frac{\tilde{\alpha_{1}}+\left( 1-\phi\right)\sum_{2}^{n-1} \tilde{\alpha_{i}}+\tilde{\alpha_{n}}}{1+\left( n-2 \right)\left( 1-\phi\right)+1}=\frac{\|\vec{g_{1}}-\vec{w}\|+\left( 1-\phi\right)\sum_{2}^{n-1} \|\vec{g_{i}}-\vec{w}\|+\|\vec{g_{n}}-\vec{w}\|}{1+\left( n-2 \right)\left( 1-\phi\right)+1}$ (4)

Using this estimator and applying Bessel's correction for sample size, i.e. multiplying by $\left[ n/\left( n-1 \right) \right]$, and assuming that $\hat{a}\approx a$, we get an estimator for the variance term:

$\begin{matrix} s^{2}\left( G,\vec{w} \right)\equiv\\ \frac{1}{n-1}\left[ \left( \tilde{\alpha_{1}}-\hat{a} \right)^{2}\left( 1-\phi^{2} \right)+\sum_{i=2}^{n} \left( \tilde{\alpha_{i}}-\left[ \left( 1-\phi\right)\hat{a}+\phi\tilde{\alpha_{i-1}} \right] \right)^{2} \right]= \\ \frac{1}{n-1}\left[ \left( \|\vec{g_{1}}-\vec{w}\|-\hat{a} \right)^{2}\left( 1-\phi^{2} \right)+\sum_{i=2}^{n} \left( \|\vec{g_{i}}-\vec{w}\|-\left[ \left( 1-\phi\right)\hat{a}+\phi\|\vec{g_{i-1}}-\vec{w}\| \right] \right)^{2} \right] \end{matrix}$ (5)

and obtain

$l\left( G,\vec{w} \right)=\frac{1}{2}\left\{ nlog\left( \sigma^{2} \right)-log\left( 1-\phi^{2} \right)+\frac{n}{\sigma^{2}}s^{2}\left( G,\vec{w} \right) \right\}$ (6)

Because the first two terms and the factor $n/{\sigma^{2}}$ are independent of $\vec{w}$, we can calculate the likelihood estimate for the unknown wind vector by minimizing the unexplained variance in air speed $s^{2}\left( G,\vec{w} \right)$:

$\hat{w}=arg\min_{\vec{w}}\left[ s^{2}\left( G,\vec{w} \right) \right]$. (7)

For each track segment, we obtain mean air speed $a$, setting $\vec{w}=\hat{w}$. Also for each segment, mean vertical speed ground speed around point *k*, $g_{z_{k}}$ is calculated as

$g_{z_{k}}=\frac{\left( z_{k+m}-z_{k-m} \right)}{\left( n-1 \right)\Delta t}$ (8)

where $z_{k}$ is GPS height at point *k*. Change in heading between two consecutive GPS fixes, $\Delta\theta_{i}$, is defined as the angle between the air speed vectors $\left( \vec{g}_{i}-\hat{w} \right)$ and $\left( \vec{g}_{k+1}-\hat{w} \right)$; the cumulative change in heading can then be defined as $\Delta\theta_{cum}\equiv\sum_{i=k-n}^{k+n-1} {\Delta\theta}_{i}$. Assuming that the bird is flying in a perfect circle at constant air speed, we calculate the mean angular rate in radians, $\dot{\omega}$, the circle radius, *r*, and the time per full circle, $\Delta t_{c}$, are:

$\dot{\omega}=\frac{\Delta\theta_{cum}}{\Delta t_{cum}};r=\frac{\dot{a}}{\dot{\omega}}; \Delta t_{c}=\frac{2\pi}{\dot{\omega}}$ (9)

Using the fact that in a balanced turn the centripetal acceleration is $\dot{a}\dot{\omega}=\left[ L/m \right]sin\left( \beta\right)$ and the gravitational acceleration $g=\left[ L/m \right]cos\left( \beta\right)$ where $L$ is the lift, $m$ the bird mass, and $\beta$ the banking angle, we obtain estimates for the banking angle

$\beta={tan}^{-1}\left( \frac{\dot{a}\dot{\omega}}{g} \right)$ (10)

and the lift acceleration

$\frac{L}{m}=\sqrt{g^{2}+\dot{a}^{2}\dot{\omega}^{2}}$ (11)


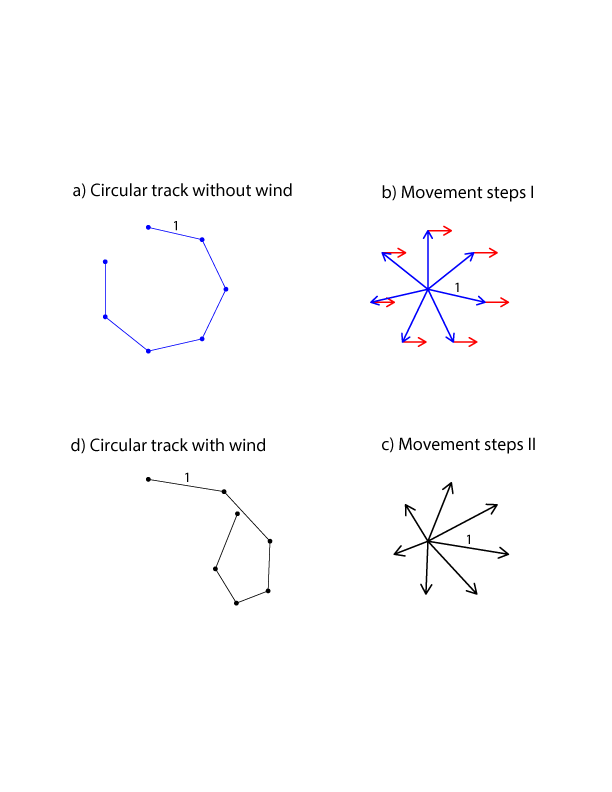


*Figure 1.Track distortion by wind. Consider positions sampled at a fixed rate from a bird which is flying in a perfect circle in windless conditions. The resulting track might look like (****a)****. Now consider* ***(b)****, which shows the vectors connecting subsequent positions from (****a)*** *(in blue). The blue vectors in* ***(b)*** *represent individual movement steps of the bird, e.g. "3m northward and 1m eastward". Now add some sidewind to the picture. The displacement of the bird in each movement step caused by sidewind is represented by the red vectors in* ***(b)****. By combining blue and red vectors we get the movement steps of a bird flying under the influence of sidewind* ***(c)****. If we append the vectors from* ***(c)*** *in chronological sequence, then we get the typical ground track of a bird which is displaced by wind while circling* ***(d)****.*
